# The impact and application exercises on vocal fatigue

**DOI:** 10.1038/s41598-026-53079-1

**Published:** 2026-05-19

**Authors:** Jing Peng, Mi Zou, Manwa L. Ng, Qiao Hou, Liping Zhu, Jie Tan, Qiannan Zhang

**Affiliations:** 1 Department of Rehabilitation, The Third Xiangya Hospital, Central South University, Changsha, China; 2 Speech Science Laboratory, Faculty of Education, The University of Hong Kong, Hong Kong, China; 3 School of Acupuncture-Moxibustion, Tuina and Rehabilitation, Hunan University of Chinese Medicine, Changsha, China; 4 Traditional Chinese Medicine School, Chongqing Three Gorges Medical College, Chongqing, China

**Keywords:** Vocal fatigue, Vocal rest, Straw phonation, Voice quality, Voice assessment, Diseases, Health care, Medical research, Neuroscience, Physiology

## Abstract

To investigate the therapeutic efficacy and application of different straw phonation exercise regimens as interventions for vocal fatigue in vocally healthy adults under simulated occupational vocal loading conditions. One hundred and fourteen vocally healthy adults aged 20–40 years with no organic lesions confirmed by fibrolaryngoscope examination were randomly assigned into the following groups: (1) control group, (2) Experimental Group A, and (3) Experimental Group B. Immediately following a one-hour vocal loading task (VLT), intervention was provided to all three groups. The control group underwent a 10-minute vocal rest, Experimental Group A performed a 10-minute straw phonation exercise, and Experimental Group B performed a 5-minute straw phonation exercise after a 5-minute vocal rest. Straw phonation consisted of sustained vowel /u:/ phonation through a straw (inner diameter: 5 mm; length: 19.5 cm), performed continuously for the assigned duration. Acoustic parameters including maximum phonation time (MPT), Fundamental frequency (F0), jitter, shimmer, harmonic-to-noise ratio (HNR) and cepstral peak prominence for speech (CPP-s) and perceptual voice assessment including Vocal Tract Discomfort (VTD) and Perceived Phonatory Effort (PPE) were performed before VLT (T0), immediately after VLT (T1), and post-intervention (T2). Data were analyzed using generalized estimating equations (GEE) to evaluate time, group, and time × group interaction effects. For acoustic voice measures, no significant changes were observed at T2 compared with T1 in the control group. Experimental Group A exhibited significant changes in MPT, F0, jitter and CPP-s at T2 compared with T1, whereas Experimental Group B demonstrated significant changes in MPT, jitter, shimmer and HNR at T2 compared with T1 (*p* < 0.05). No differences were observed among the three groups at T0. At T2, Experimental Group B showed superior jitter, shimmer, HNR, and CPP-s values compared with the control group, while Experimental Group A had higher CPP-s values than the control group. Additionally, Experimental Group B had higher HNR compared with Experimental Group A . For subjective voice measures, both Experimental Groups A and B outperformed the control group in the VTD (lump in the throat) and PPE scores at T2, and Experimental Group A had higher scores of VTD (Total) than Experimental Group B. Straw phonation produced greater short-term improvements than vocal rest alone in acoustic and subjective voice measures. After prolonged vocal use, a 5-minute vocal rest followed by 5-minute straw phonation yielded greater immediate voice quality improvements and reduced vocal tract discomfort compared with 10-minute straw phonation training, illustrating the importance of timing in the clinical application of straw phonation after prolonged phonatory loading tasks as indicated by objective and subjective voice measures.

*Trial registration: *This study was registered in the Ethics Committee of the Third Xiangya Hospital of Central South University (Protocol no. 24508) on 2024-06-14, and approved by the Chinese Clinical Trial Registry (ChiCTR2400087198) on 2024-07-22. This trial record can be accessed on the official ChiCTR website https://www.chictr.org.cn/bin/project/edit?pid=232,332.

## Introduction

Voice disorders, known as dysphonia, often manifest as fatigue, pain, dryness of the throat, laryngeal muscle tension, or vocal weakness following prolonged phonation^1^. Among these, vocal fatigue is one of the most common complaints in individuals with dysphonia^1^. Although vocal rest may partially alleviate these symptoms, compensatory mechanisms associated with vocal fatigue have been observed and may increase the risk of vocal fold damage and laryngeal pathologies^2^. These include inefficient extrinsic laryngeal muscle co-activation, glottal malpositioning with excessive medial compression during phonation, and compensatory hyperactivation of extrinsic laryngeal musculature^3^. Consequently, vocal fatigue remains a significant public health concern, especially for professional voice users such as teachers, lawyers, and personal trainers^4^.

Traditional rest-based approaches for the management of vocal fatigue are increasingly challenged^1^. Occupational or professional voice users often lack sufficient rest time, although higher fatigue levels necessitate longer recovery periods^5^. Studies have indicated that only approximately 35% of vocal fatigue patients fully adhere to prescribed voice rest, as suggested by speech therapists, significantly compromising treatment efficacy^6,7^. Furthermore, following a one-hour vocal loading task (VLT), it generally requires at least 20 min for the voice to return to baseline levels, as indicated by various acoustic, aerodynamic, and perceptual measures^8^. However, professional voice users, such as teachers, often cannot afford to rest their voice for a period of time, even as short as 10 min, resulting in a failure to achieve full recovery. Consequently, their non-stop intensive teaching renders further vocal discomfort, thereby exacerbating the risk of vocal fold injury.

Straw phonation, a semi-occluded vocal tract (SOVT) exercise, is widely practiced in clinical voice therapy^9^. Research has confirmed that VLT can induce dysphonia due to vocal fold edema^10^. Straw phonation is a cyclic physiological vocal exercise that provides tensile strain at low physiological magnitudes to the vocal fold tissues^11^. By semi-occluding the vocal tract at the lips, straw phonation increases supraglottal pressure and vocal tract inertive reactance, thereby strengthening source–filter interaction, lowering phonation threshold pressure, and attenuating vocal fold collision stress, which together enhance vocal efficiency and reduce phonatory effort during prolonged voice use^12^. In addition, the slightly positive intraoral pressure during straw exercises may promote extracellular matrix synthesis under mild cyclic tension, reducing inflammation and facilitating tissue repair^12–14^. Through these combined biomechanical and aerodynamic mechanisms, straw phonation has been suggested to leverage the body’s natural response to stress and alleviate vocal fatigue^15^.

However, whether these physiological benefits translated to simulated occupational vocal loading conditions remains unclear, particularly under prolonged voice use resembing teaching schedules. Accordingly, it remains unclear whether professional voice users, such as teachers, can tolerate an additional 10 min of straw phonation during short breaks after prolonged teaching sessions or whether combining rest with this training outperforms rest or training alone. Therefore, this study aimed to investigate the effects of different regimens for straw phonation and its combination with vocal rest on vocal fatigue in healthy adults simulated occupational vocal loading conditions. Both objective acoustic parameters and subjective voice measurements were assessed, thereby providing evidence-based insights for optimizing therapeutic protocols for professional voice users such as teachers.

## Methods

### Study design and ethical approval

This prospective randomized experimental study was approved by the Ethics Committee of the Third Xiangya Hospital of Central South University (Protocol no.: 24508) on 2024-06-14, and registered with the Chinese Clinical Trial Registry (ChiCTR2400087198) on 2024-07-22. This trial record can be accessed on the official ChiCTR website: https://www.chictr.org.cn/bin/project/edit?pid=232332. Written informed consent was obtained from all participants before the experiment began.

### Participants

This study recruited 114 vocally healthy non-professional voice users between September 2024 and December 2024 from The Third Xiangya Hospital of Central South University. All participants were confirmed to have no organic lesions via fibrolaryngoscopy under continuous light, performed by an experienced otolaryngologist. Only participants who met the following criteria were recruited: (1) aged 20–40 years, (2) no history of smoking, (3) no alcohol-related health complications, (4) no voice complaints or history of voice disorders, (5) absence of acute infections within two weeks prior to participation, and (6) avoidance of vocal overuse (e.g., singing, shouting, or loud speaking), spicy foods, carbonated/caffeinated beverages, and alcohol for 24 h before the study. In addition, the participants were instructed to refrain from eating for one hour before the experiment to prevent laryngopharyngeal reflux.

The exclusion Criteria were as follows: (1) any organic vocal fold lesions; (2) respiratory, neurological, cardiovascular, or hepatic diseases; and (3) cognitive impairments, psychiatric disorders, or conversion disorders.

### Protocol

This study was a prospective randomized experimental study. Prior to participant enrollment, 114 random numbers were sequentially selected from a standard random number table to generate the allocation sequence. Each number was divided by three, and participants were assigned according to the remainder (0, 1, or 2), corresponding to the control group, Experimental Group A, and Experimental Group B, respectively, resulting in equal allocation (1:1:1), with 38 participants assigned to each group. The allocation sequence was generated in advance by a researcher who was not involved in participant recruitment, intervention delivery, or outcome assessment. Allocation concealment was ensured using sequentially numbered, sealed, opaque envelopes, which were opened only after completion of baseline assessment. Participant enrollment and intervention delivery were conducted by the speech-language therapist based on the group assignments revealed after envelope opening. One day prior to the experiment, all participants received a brief standardized familiarization session with straw phonation, including demonstration, short supervised practice, and a standard instructional video, to ensure correct technique without delivering a therapeutic dose or affecting baseline measures. Participants were unaware of their group allocation until the intervention began. Due to the nature of the intervention, the speech-language therapist was aware of group allocation. However, the statistician responsible for data analysis was blinded to group assignment throughout the statistical procedures.

All participants completed a one-hour VLT, followed by a 10-minute intervention (vocal rest only, straw phonation only, or a combination of vocal rest and straw phonation). The control group underwent a 10-minute vocal rest; Experimental Group A performed a 10-minute straw phonation; and Experimental Group B completed a 5-minute vocal rest followed by a 5-minute straw phonation.

**Fig. 1:**
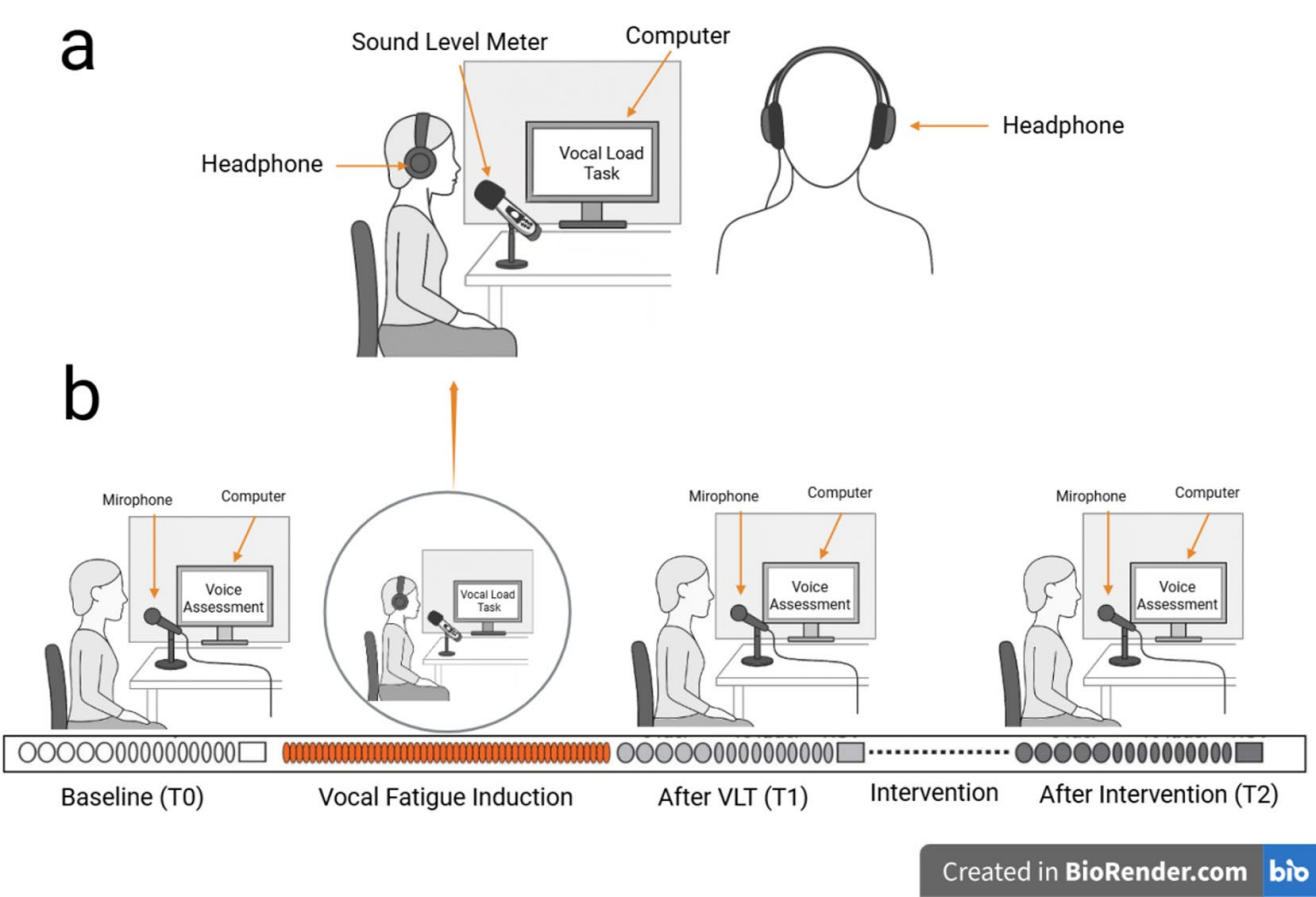
Experimental design and procedures. **a** Diagram illustrating the experimental setup for the acquisition of voice assessment and Vocal Load Task. **b** Diagram representing the different stages of the experimental session. Utterances and procedures are illustrated for each experimental condition. *VLT: * Vocal Loading Task.

The primary outcome measures, as prespecified in the trial registration, included objective acoustic parameters: the maximum phonation time (MPT), fundamental frequency (F0), jitter, shimmer, harmonic-to-noise ratio (HNR), cepstral peak prominence (CPP) and the Vocal Tract Discomfort (VTD) scale^16^. The Perceived Phonatory Effort (PPE) scale^17^ was a secondary outcome measure. The VTD scale has demonstrated good reliability and validity across multiple validated language versions in previous studies^16^. As no validated Chinese version of the VTD scale is currently available, a Chinese translation was prepared by the research team for standardized administration and administered uniformly to all participants. Clear written instructions were provided prior to completion. All measurements were obtained three times to evaluate changes in vocal function across different phases: before VLT as baseline (T0), immediately after VLT (T1), and immediately after intervention (T2). An overview of the experiments is shown in Fig. 1.

### Vocal loading task (VLT)^18^

VLT is a widely used method for inducing vocal fatigue. In the present study, participants were instructed to read aloud a standardized novel story for 60 min, maintaining a volume of 75–80 dB, as measured by a digital sound level meter (SMART SENSOR, AR844) positioned 40 cm from the lips. Background noise (environmental and human speech) was delivered via headphones at 75 dB and calibrated using a sound-level meter prior to the trial. Participants were allowed a 30-second break every 15 min and provided with 100 mL of standardized room-temperature mineral water to maintain hydration and minimize potential confounding effects.

### Vocal fatigue management

To simulate a typical teaching schedule of 60-minute lectures followed by 10-minute breaks, the intervention duration was set at 10 min. The straw phonation protocol was based on semi-occluded vocal tract (SOVT) techniques described by Titze^19^. Standardized instructional guidance was adapted from the instructional video “Ingo Titze’s Tips for a Tired Voice: Grab a Straw!” (Titze, I.n.d. *Ingo Titze’s tip for tired voices: Grab a straw!* YouTube). Retrieved February 10, 2024 (https://www.youtube.com/watch?v=0xYDvwvmBIM). The participants were required to review this video for standardized technique training. Training Specifications: (1) Straw dimension: 19.5 cm in length with a 5 mm inner diameter; (2) Pre-trial preparation: ensure airtight lip-straw seal; and (3) Phonation execution: adopt abdominal breathing to maintain steady expiratory airflow; avoid excessive extrinsic laryngeal muscle activation, and sustain the vowel/u:/at a steady pitch and intensity. And participants were instructed to sustain the vowel/u:/continuously throughout the assigned duration, allowing natural inhalation between phonation cycles without structured rest intervals. All sessions were monitored and supervised by a practicing speech therapist to ensure correct straw placement, an adequate lip-straw seal, appropriate breathing pattern, and stable phonation. In cases where participants were unclear or did not understand, feedback and guidance were provided by professional speech therapists immediately to maintain technical fidelity. Adherence was monitored throughout the session to promote procedural consistency and reproducibility across participants. During the vocal rest sessions, the participants were instructed to maintain complete vocal silence for the designated duration (10 min for the control group and 5 min for Experimental Group B).

### Data collection

Objective and subjective voice assessments were carried out at three time points: pre-VLT (baseline) (T0), immediately after VLT (T1), and immediately after intervention (T2). Acoustic analysis was performed using Praat software (version 5.4.22) to obtain the following acoustic measures: MPT, F0, percent jitter, percent shimmer, HNR, and CPP for continuous speech (CPP-s). CPP-s was calculated using a dedicated CPP plugin developed by Murray, Chao, and Colletti^20^.

During the experiment, participants completed three speech tasks: (1) sustained phonation of the vowel /a/ for ≥ 5 s; (2) prolongation of the vowel /a/ maximally, repeated three times; and (3) oral reading of a 30-word standardized Chinese passage at natural loudness and pitch.

All the recordings were conducted in a soundproof booth with a constant ambient noise of 30–35 dB. A condenser microphone (Alctron CU48) was positioned 10 cm from the participant’s mouth and connected to a preamplifier (M-Audio PreUSB) and PC. Acoustic signals were sampled at 44.1 kHz with 16-bit/sample quantization. Acoustic parameters were extracted using Praat software (version 5.4.22) with identical analysis settings applied across all recordings. Measurements were obtained using automated procedures without manual intervention; therefore, intra- or inter-rater reliability was not applicable.

Subjective voice assessment, including VTD and PPE, was obtained by reading Chinese short passages aloud. PPE was evaluated using a visual analog scale (VAS), with a score of 0 indicating no effort at all and a score of 10 indicating extremely effortful production. The participants were also rated on the basis of their perceived effort during phonation (PPE) using a VAS. The VTD scale provides a useful perceptual indicator of sensory changes^21^ and previous studies have investigated the relationship between VTD and vocal load activity^22^. VTD indicates the frequency with which they occur and the severity of the symptom/sensation. Given that all the subjects involved in this study were healthy adults, only the severity of the VTD scale was selected to measure different phases of the study. The VTD evaluated eight symptoms or sensations that can be felt in the throat (i.e., burning, tightness, dryness, aching, tickling, sore, irritable, and lump in the throat). All participants were required to rate the severity of each symptom on a seven-point Likert scale (0–6), ranging from no discomfort to severe perception, with higher scores indicating greater vocal tract discomfort^23^. Written instructions were also provided. Lump sensation in the throat is a high-frequency symptom of VLT^22^ and better reflects the subjective perception of vocal fold viscosity^24^. Therefore, this study also analyzed the sub-item of “lump in the throat” independently in addition to calculating the VTD total score.

### Statistical analysis

#### Sample size calculation

The sample size was initially calculated using the G*Power software (v3.1.9.7), assuming an effect size (Cohen’s *f*) of 0.2, significance level of 0.05, 95% power, three groups, three repeated measurements under the “ANOVA repeated measures, within-between interaction” model. The calculation was based on a standardized effect size rather than raw mean differences, no specific standard deviation was entered. The calculation yielded a required samples of 27 individuals per group; therefore, the sample size was estimated to be 81 participants, with an additional 20% (*n* = 18) allocated to account for potential attrition, resulting in a planned enrollment of 99 participants.

#### Statistical methods

Statistical analysis of the data was performed using SPSS 27. To ensure the retention of all randomized participants, avoid bias, and reflect the “real-world effectiveness” of the intervention, analyses followed the intention-to-treat principle. For missing follow-up data from participants who withdrew midway or failed to complete the trial as required, the last observation carried forward (LOCF) method was used.

Generalized estimating equations (GEE) were employed for data of MPT, F0, jitter, shimmer, HNR, CPP-s, VTD (lump in the throat), VTD (Total) and PPE across time points and groups. Main effects of group and time, as well as the group × time interaction, were tested. When GEE showed significant effects (*P* < 0.05), Bonferroni-adjusted pairwise comparisons were conducted within the GEE framework to examine within-group changes over time and between-group differences at each time point. Model-based results are presented as estimated marginal means (EMMs) with 95% confidence intervals for each group at each time point, and as estimated mean differences with 95% confidence intervals for post-hoc comparisons. All statistical tests were two-sided. A two-sided *P* < 0.05 was considered statistically significant for the GEE model, and Bonferroni-adjusted *P* < 0.05 was considered statistically significant for post-hoc comparisons.

## Results

**Fig. 2:**
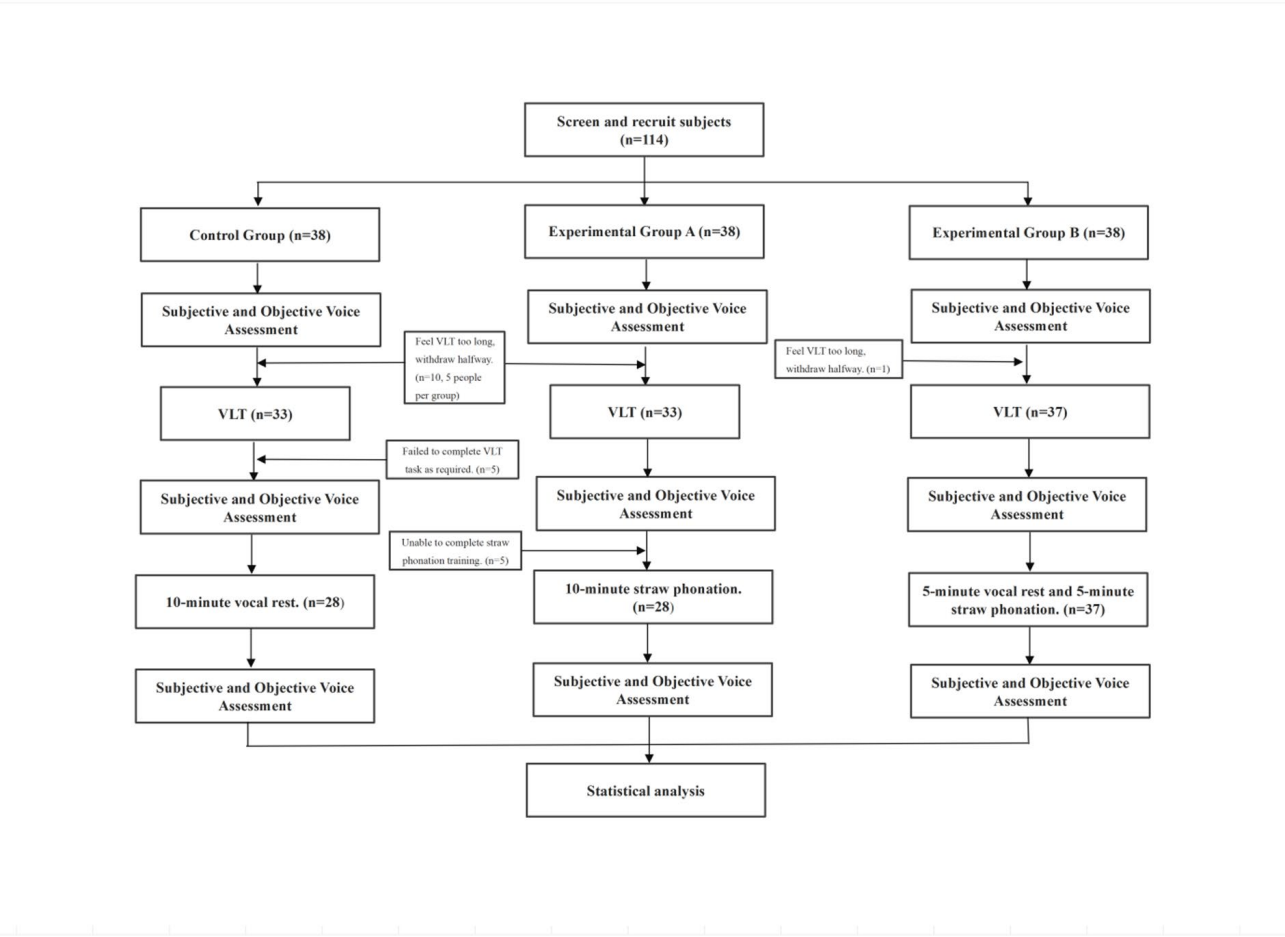
Flowchart of Research. *VLT:* Vocal Loading Task.

A total of 114 participants were initially enrolled, as shown in Fig. 2. The initial cohort comprised 38 (9 males, 29 females, aged 23.84 ± 4.940 years) in the Control Group, 38 (6 male, 32 females, aged 22.11 ± 3.367 years) in Experimental Group A, and 38 participants (6 male, 32 females, aged 22.26 ± 3.957 years) in Experimental Group B. There were no between-group differences in age and gender (*p* > 0.05), and baseline assessment parameters (*p* > 0.05 for all comparisons; see Table 3). Of the 114 randomized participants, 93 (81.6%) completed the trial, while 21 participants (18.4%) withdrew during the study (Control Group: *n* = 10; Experimental Group A: *n* = 10; Experimental Group B: *n* = 1). Missing follow-up data from these participants were handled using the LOCF method. The reasons for withdrawal are presented in Fig. 2.

### Voice assessment

Regarding MPT, significant main effects were found for the assessment time (Wald χ² = 14.902, *p* = 0.001; Table 1). Post-hoc analyses showed improvements in MPT over time within the experimental groups (Table 2). Between-group comparisons indicated numerically higher MPT values in Experimental Group B at T2 compared with the control group, but the difference did not reach statistical significance.

**Fig. 3:**
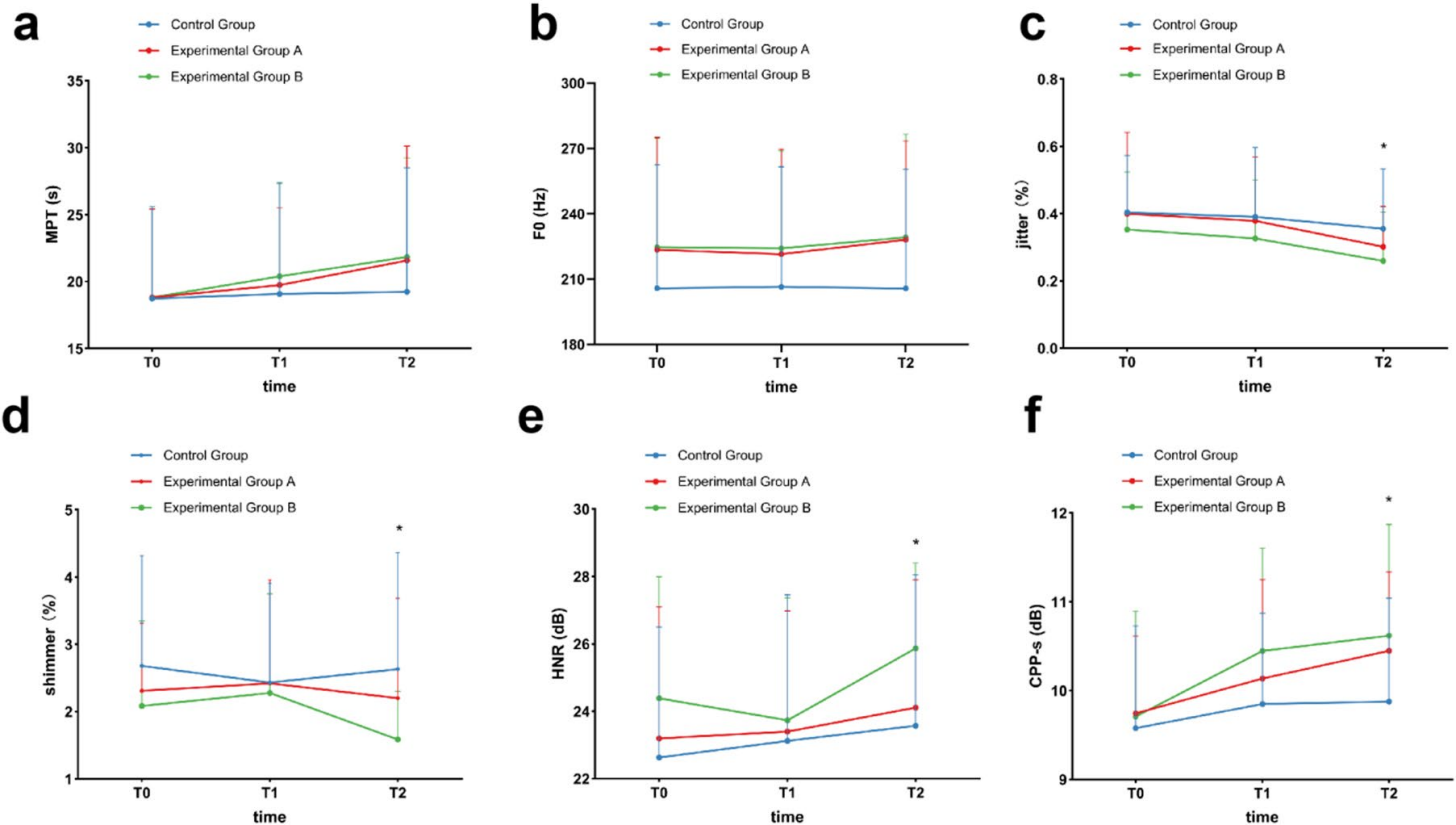
Between-Group Comparison of Vocal Acoustic Parameters. *Comparisons with significant results; *MPT:* maximum phonation time, *HNR:* harmonics-to-noise ratio, *CPP-s:* cepstral peak prominence for speech, *T0:* baseline level, *T1:* after the vocal loading task, *T2:* after intervention.

The results indicated significant main effects of time for F0 (Wald χ² = 6.743, *p* = 0.034; Table 1). Experimental Group A showed significant within-group differences across time (Table 2). Between-group comparisons at each time point also revealed no significant differences among the three groups (Table 3; Fig. 3).

The results of the jitter showed a significant main effect of time (Wald χ² = 33.648, *p* < 0.001; Table 1). Temporal changes were observed in three groups (Table 2). Notably, lower jitter values were found in Experimental Group B compared with the control group at T2 (0.260 [95% CI: 0.214, 0.305] vs. 0.356 [95% CI: 0.300, 0.411]; Table 3; Fig. 3).

Significant differences were found for shimmer in the main effect of time (Wald χ² = 5.242, *p* = 0.024) and the interaction between condition and time (Wald χ² = 11.483, *p* = 0.008), as shown in Table 1. Within-group comparisons demonstrated temporal changes in Experimental Group B (Table 2). Furthermore, shimmer values were significantly lower in Experimental Group B compared with the control group at T2 (1.586 [95% CI: 1.361, 1.811] vs. 2.632 [95% CI: 2.090, 3.175]; Table 3; Fig. 3).

Connecting HNR, the results revealed a significant main effect of time (Wald χ² = 26.118, *p* < 0.001; Table 1). HNR in control group and Experimental Group B showed temporal changes (Table 2). Between-group comparisons showed significantly higher HNR values in Experimental Group B compared with the control group and Experimental Group A at T2 (25.868 [95% CI: 25.076, 26.661] vs. 23.574 [95% CI: 22.169, 24.978]; 25.868 [95% CI: 25.076, 26.661] vs. 24.111 [95% CI: 22.922, 25.301]; Table 3; Fig. 3).

In terms of CPP-s, significant main effects of time (Wald χ² = 73.612, *p* < 0.001) and interaction between condition and time (Wald χ² = 12.992, *p* = 0.011) were observed (Table 1). CPP-s showed temporal changes in both Experimental Groups A and B (Table 2). Between-group comparisons demonstrated significantly higher CPP-s values in Experimental Group A and Experimental Group B compared with the control group at T2 (10.449 [95% CI: 10.172, 10.727] vs. 9.878 [95% CI: 9.512, 10.243]; 10.620 [95% CI: 10.227, 11.013] vs. 9.878 [95% CI: 9.512, 10.243]; Table 3; Fig. 3).

**Fig. 4:**
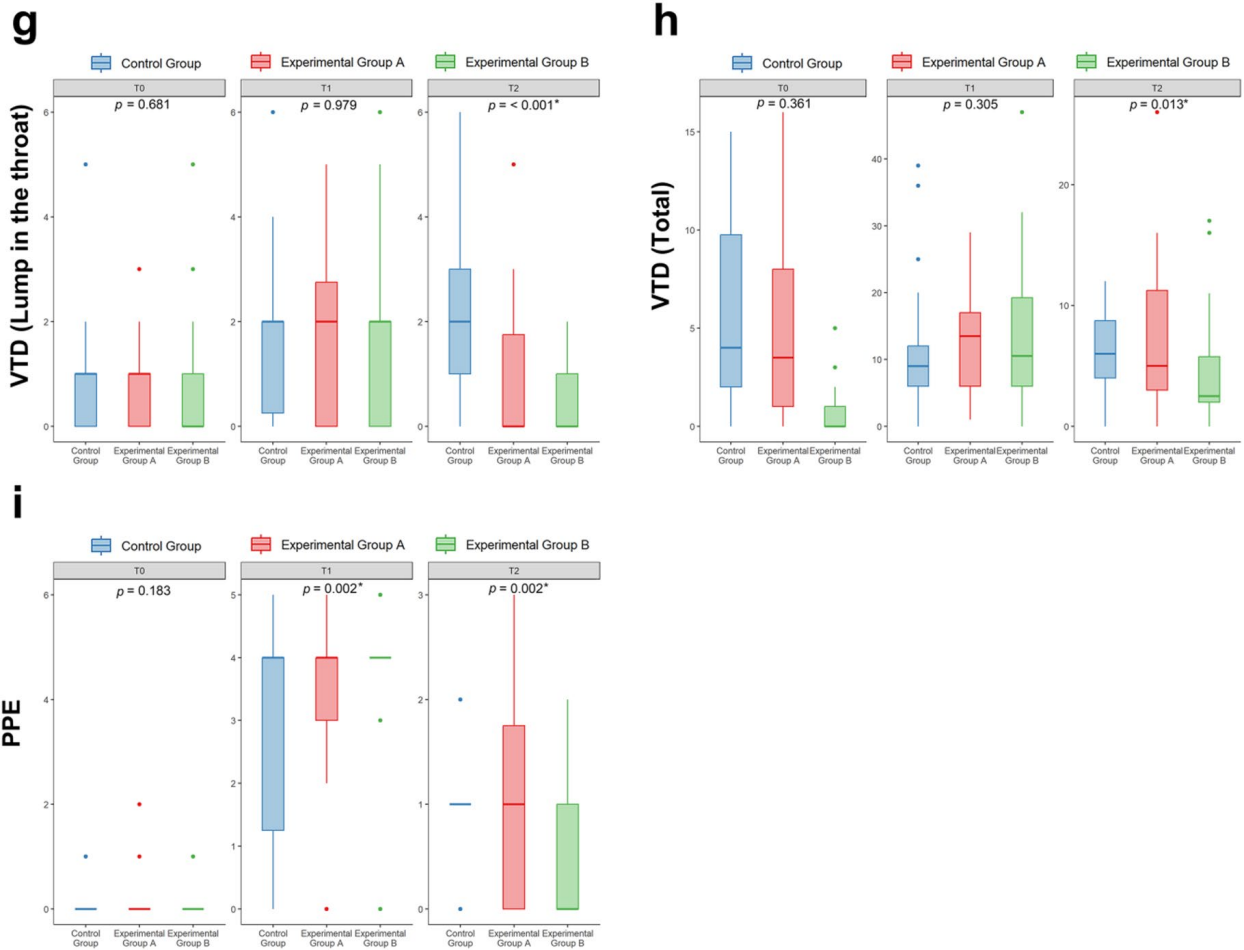
Between-Group Comparison of Subjective Voice Assessment. *p* values indicate the omnibus between-group difference at each time point based on the GEE Wald χ² test. *Comparisons with significant results; *VTD:* vocal tract discomfort, *PPE:* perceived phonatory effort, *T0:* baseline Level, *T1:* after the vocal loading task, *T2:* after intervention.

With regard to VTD (lump in the throat), significant main effects of condition (Wald χ² = 6.235, *p* = 0.044), time (Wald χ² = 47.976, *p* < 0.001), and their interaction (Wald χ² = 33.563, *p* < 0.001) were found (Table 1). Within-group temporal changes were observed across all groups (Table 2). Between-group comparisons indicated significantly lower VTD (lump in the throat) scores in Experimental Groups A and B compared with the control group at T2 (0.89 [95% CI: 0.52, 1.27] vs. 1.92 [95% CI: 1.50, 2.34] and 0.50 [95% CI: 0.30, 0.70] vs. 1.92 [95% CI: 1.50, 2.34]; Table 3; Fig. 4).

With respect to the VTD (total), a significant main effect of time was observed (Wald χ² = 102.283, *p* < 0.001; Table 1). Temporal changes within groups were shown in Table 2. At T2, Experimental Group B exhibited significantly lower total VTD scores compared with both the control group and Experimental Group A (4.11 [95% CI: 2.83, 5.38] vs. 6.18 [95% CI: 5.11, 7.26] and (4.11 [95% CI: 2.83, 5.38] vs. 7.00 [95% CI: 5.16, 8.84], Table 3; Fig. 4).

Regarding PPE, significant main effects of time (Wald χ² = 528.463, *p* < 0.001) and interaction between condition and time (Wald χ² = 43.928, *p* < 0.001) were observed (Table 1). PPE showed temporal change within all groups (Table 2). Between-group comparisons demonstrated significantly higher PPE values in Experimental Group B compared with the control group at T1 (3.87 [95% CI: 3.61, 4.12] vs. 2.89 [95% CI: 2.34, 3.45], and lower PPE values in Experimental Groups A and B compared with the control group at T2 (0.58 [95% CI: 0.38, 0.78] vs.1.00 [95% CI: 0.78, 1.22]; 0.50 [95% CI: 0.28, 0.72] vs. 1.00 [95% CI: 0.78, 1.22]; Table 3; Fig. 4).

**Table 1:** Generalized estimating equation (GEE) results for the main and interaction effects of time and condition in objective and subjective parameters.

Source of Variation	MPT (s)		F0 (Hz)		Jitter (%)		Shimmer (%)		HNR (dB)	
	Wald	*P*	Wald	*P*	Wald	*P*	Wald	*P*	Wald	*P*
Condition	0.786	0.675	3.601	0.165	4.764	0.092	5.510	0.064	5.019	0.081
Time	14.902	0.001^*^	6.743	0.034^*^	33.648	< 0.001^*^	5.242	0.024^*^	26.118	0.000^*^
Condition × time	4.984	0.289	4.839	0.304	3.852	0.426	11.483	0.008^*^	9.293	0.054

Source of Variation	CPP-s (dB)		VTD (lump in the throat)		VTD (total)		PPE			
	Wald	*P*	Wald	*P*	Wald	*P*	Wald	*P*		
Condition	4.674	0.097	6.235	0.044^*^	1.432	0.489	3.416	0.181		
Time	73.612	< 0.001^*^	47.976	< 0.001^*^	102.283	< 0.001^*^	528.463	< 0.001^*^		
Condition × time	12.992	0.011^*^	33.563	< 0.001^*^	8.515	0.074	43.928	< 0.001^*^		

Wald χ² statistics from Generalized estimating equation (GEE) models. ^*^P < 0.05. *MPT:* maximum phonation time, *HNR:* harmonic-to-noise ratio, *CPP-s:* cepstral peak prominence for speech, *VTD:* vocal tract discomfort, *PPE:* perceived phonatory effort, *T0:* baseline level, *T1:* after the vocal loading task, *T2:* after intervention.

**Table 2:** Within-Group Comparisons of Objective and Subjective Parameters.

Dependent Variables	Time	Control Group		Experimental Group A		Experimental Group B	
		Mean Difference	*P*	Mean Difference	*P*	Mean Difference	*P*
MPT (s)	T1-T0	0.340	1.000	0.920	1.000	1.580	1.000
	T2-T0	0.500	1.000	2.760	0.004^*^	3.030	< 0.001^*^
	T2-T1	0.160	1.000	1.840	0.034^*^	1.450	0.034^*^
F0 (Hz)	T1-T0	0.707	1.000	−1.980	1.000	−0.501	1.000
	T2-T0	−0.044	1.000	4.651	1.000	4.493	0.703
	T2-T1	−0.751	1.000	6.632	0.012^*^	4.999	0.179
Jitter (%)	T1-T0	−0.013	1.000	−0.021	1.000	−0.027	1.000
	T2-T0	−0.048	0.036^*^	−0.098	0.343	−0.094	< 0.001^*^
	T2-T1	−0.035	0.458	−0.077	0.001^*^	−0.067	0.033*
Shimmer (%)	T1-T0	−0.247	1.000	0.111	1.000	0.194	1.000
	T2-T0	−0.048	1.000	−0.110	1.000	−0.499	0.017^*^
	T2-T1	0.199	0.788	−0.221	0.537	−0.693	0.001^*^
HNR (dB)	T1-T0	0.496	0.734	0.205	1.000	−0.653	0.912
	T2-T0	0.942	0.022^*^	0.913	0.473	1.482	0.008^*^
	T2-T1	0.447	0.752	0.708	0.352	2.135	< 0.001^*^
CPP-s (dB)	T1-T0	0.272	0.143	0.393	0.025^*^	0.740	< 0.001^*^
	T2-T0	0.299	0.135	0.704	< 0.001^*^	0.912	< 0.001^*^
	T2-T1	0.027	1.000	0.312	0.002^*^	0.172	0.606
VTD (lump in the throat)	T1-T0	0.840	0.003^*^	1.000	< 0.001^*^	1.000	< 0.001^*^
	T2-T0	1.030	< 0.001^*^	0.130	1.000	−0.180	0.763
	T2-T1	0.180	0.933	−0.870	< 0.001^*^	−1.180	< 0.001^*^
VTD (total)	T1-T0	5.260	< 0.001^*^	7.790	< 0.001^*^	9.370	< 0.001^*^
	T2-T0	0.920	0.295	1.820	0.121	0.160	1.000
	T2-T1	−4.340	< 0.001^*^	−5.970	< 0.001^*^	−9.210	< 0.001^*^
PPE	T1-T0	2.710	< 0.001^*^	3.110	< 0.001^*^	3.760	< 0.001^*^
	T2-T0	0.820	< 0.001^*^	0.530	< 0.001^*^	0.390	0.039^*^
	T2-T1	−1.890	< 0.001^*^	−2.580	< 0.001^*^	−3.370	< 0.001^*^

Pairwise time contrasts were derived from generalized estimating equations (GEE), with Bonferroni adjustment. *P < 0.05. *MPT:* maximum phonation time, *HNR:* harmonic-to-noise ratio, *CPP-s:* cepstral peak prominence for speech, *VTD:* vocal tract discomfort, *PPE:* perceived phonatory effort, *T0:* baseline level, *T1:* after the vocal loading task, *T2:* after intervention.

**Table 3:** Between-Group Comparisons of Objective and Subjective Parameters.

Dependent Variables	Time	Control Group EMM (95% CI)	Experimental Group A EMM (95% CI)	Experimental Group B EMM (95% CI)
MPT (s)	T0	18.74 (16.58, 20.89)	18.82 (16.74, 20.89)	18.82 (16.74, 20.89)
	T1	19.08 (16.47, 21.69)	19.74 (17.93, 21.54)	20.39 (18.23, 22.56)
	T2	19.24 (16.33, 22.14)	21.58 (18.90, 24.26)	21.84 (19.52, 24.17)
F0 (Hz)	T0	205.789 (187.971, 223.604)	223.434 (207.220, 239.648)	224.716 (209.066, 240.367)
	T1	206.495 (189.207,223.783)	221.454 (206.290,236.618)	224.210 (210.178,238.241)
	T2	205.744 (188.569,222.918)	228.086 (213.870,242.301)	229.209 (214.384,244.034)
Jitter (%)	T0	0.404 (0.351, 0.457)	0.400 (0.324, 0.476)	0.353 (0.300.0.407)
	T1	0.391 (0.326, 0.455)	0.379 (0.319, 0.438)	0.326 (0.272,0.381)
	T2	0.356 (0.300, 0.411)	0.302 (0.264, 0.339)	0.260 (0.214,0.305)^b^
Shimmer (%)	T0	2.680 (2.165, 3.195)	2.311 (1.996, 2.625)	2.085(1.688,2.482)
	T1	2.433 (1.971, 2.896)	2.422 (1.938, 2.905)	2.279(1.817,2.741)
	T2	2.632 (2.090,3.175)	2.200 (1.735, 2.666)	1.586(1.361,1.811)^b^
HNR (dB)	T0	22.631 (21.417, 23.846)	23.198 (21.975,24.422)	24.387 (23.253,25.521)
	T1	23.127 (21.769, 24.485)	23.403 (22.283,24.523)	23.733 (22.594,24.823)
	T2	23.574 (22.169, 24.978)	24.111 (22.922,25.301)	25.868 (25.076,26.661)^bc^
CPP-s (dB)	T0	9.578 (9.217, 9.939)	9.745 (9.471, 10.019)	9.708 (9.337, 10.080)
	T1	9.850 (9.531, 10.170)	10.138 (9.788, 10.487)	10.448 (10.085,10.811)
	T2	9.878 (9.512, 10.243)	10.449 (10.172, 10.727)^a^	10.620 (10.227,11.013)^b^
VTD (lump in the throat)	T0	0.89 (0.57, 1.22)	0.76 (0.50, 1.03)	0.68 (0.33, 1.04)
	T1	1.74 (1.28, 2.20)	1.76 (1.30, 2.23)	1.68 (1.10, 2.27)
	T2	1.92 (1.50, 2.34)	0.89 (0.52, 1.27)^a^	0.50 (0.30, 0.70)^b^
VTD (total)	T0	5.26 (3.94, 6.58)	5.18(3.60, 6.77)	3.95 (2.50, 5.39)
	T1	10.53 (7.87, 13.19)	12.97 (10.62, 15.33)	13.32 (10.02, 16.61)
	T2	6.18 (5.11, 7.26)	7.00 (5.16, 8.84)	4.11 (2.83, 5.38)^bc^
PPE	T0	0.18 (0.06, 0.31)	0.05 (−0.02, 0.12)	0.11 (0.01, 0.20)
	T1	2.89 (2.34, 3.45)	3.16 (2.60, 3.72)	3.87 (3.61, 4.12)^b^
	T2	1.00 (0.78, 1.22)	0.58 (0.39, 0.77)^a^	0.50 (0.28, 0.72)^b^

Between-Group comparisons were performed within the GEE framework. Values are presented as estimated marginal means (EMMs) with 95% confidence intervals (CIs), and between-group difference are reported as estimated mean differences with 95% CIs. *P* values were values were Bonferroni-adjusted. ^*^P < 0.05. a: Experimental Group A vs. control group, *P* < 0.05; b: Experimental Group B vs. control group, *P* < 0.05; c: Experimental Group A vs. Experimental Group B, *P* < 0.05; *MPT:* maximum phonation time, *HNR:* harmonic-to-noise ratio, *CPP-s:* cepstral peak prominence for speech, *VTD:* vocal tract discomfort, *PPE:* perceived phonatory effort, *T0:* baseline level, *T1:* after the vocal loading task, *T2:* after intervention.

## Discussion

The Vocal Loading Task (VLT) has been used as a standardized protocol to induce vocal fatigue under controlled conditions. It is widely employed in laboratory settings to investigate voice-use patterns in healthy individuals^18^. Yet, outcomes of VLT have been found to be influenced by a number of external factors (e.g., background noise)^25^ and internal factors (e.g., vocal intensity and duration)^18^. Acoustic, aerodynamic, and perceptual metrics are commonly used to assess post-VLT voice quality^26^. Although prolonged loud reading in noisy environments (such as over 2 h) can induce vocal changes, such VLT protocols lack clinical feasibility^27^. Shorter tasks lasting for less than 30 min often fail to elicit sustained vocal alterations due to the resilience of healthy vocal mechanisms^28^, accompanied by high interspeaker variations. To simulate typical teaching schedules and vocal loading of teachers, the present study implemented a 1-hour loud speaking task under background noise. This was used to simulate the actual classroom teaching scenario faced by the teacher every day. The temporal patterns observed across parameters suggest that the VLT primarily induced transient physiological and perceptual changes at T1, whereas the interventions influenced early recovery responses at T2. Prolonged phonation alters vocal fold fluid dynamics, increasing mucosal viscosity, causing “lump in the throat” sensation, and reducing vocal efficiency^11^. Consequently, the “lump in the throat” subscale of the Vocal Tract Discomfort (VTD) scale was used independently to evaluate intervention efficacy.

The MPT reflects the interaction between expiratory airflow control and glottal impedance toward the outward airstream during phonation^29^. Although the one-hour VLT successfully induced vocal fatigue and increased MPT in the experimental groups at T2 compared with T1 in the experimental groups. This phenomenon suggested that straw phonation facilitates short-time post-loading recovery more efficiently than vocal rest alone. Straw phonation has been hypothesized to improve phonation by adjusting phonatory aerodynamics^1^. During training, the distal portion of the vocal tract is closed, leading to increased supraglottic pressure and decreased transglottic pressure, thereby enhancing source–filter interaction and increasing vocal tract inertance^30^. However, no significant differences in MPT values were observed among groups at T2. This suggests that straw phonation may promote more efficient recovery from vocal loading rather than altering overall phonatory capacity within a short intervention period. MPT is commonly considered a functional indicator of vocal endurance and phonatory efficiency during sustained speech. The faster recovery of MPT observed in the experimental groups may indicate that straw phonation facilitates more efficient restoration of vocal function after vocal loading. This effect may be particularly important for professionals such as teachers who are frequently exposed to prolonged vocal demands. In addition, post-VLT MPT values associated with the three groups remained unchanged at T1 compared with T0. This finding is in contrast to retrospective studies, as reported by Eustace et al.^31^ and D’haeseleer et al.^32^, in which reduced MPT in VF patients without organic laryngeal lesions was observed. It should be noted that these studies involved patients with confirmed vocal fatigue, whereas the participants in the current study were healthy adults who completed one-hour of VLT, which might explain the differences in findings.

Fundamental frequency (F0) describes the rate of vocal fold vibration, and it is correlated with the length, mass, and tension of the vocal folds, as well as the subglottal pressure^33^. Due to shorter vocal folds in females, F0 is inherently higher in females, which is about twice as high as that in males^34^. This study included a larger proportion of female participants and a smaller proportion of male participants with no separate analysis conducted by gender. Analyses revealed no significant F0 changes across groups at T0 and T1, indicating that VLT did not alter F0. This possibly indicates that the key vocal fold properties (length, mass, and tension) were not greatly altered and remained relatively intact. These findings align with those of Neils et al.^35^, although increased post-VLT F0 has been reported in some studies^36^. These discrepancies are potentially due to the heterogeneous cohorts and inconsistent vocal dose metrics used^18^. Interestingly, F0 increased in Experimental Group A at T2 compared with T1, whereas there was no difference in Experimental Group B. We speculate that continuous 10-minute straw phonation could provide sustained supraglottic pressure, improving source-filter interaction, thereby facilitating post-loading recalibration of phonation and reaching the dose threshold required to elicit a measurable increase in F0^19^. In contrast, the shorter 5-minute straw phonation intervention in Experimental Group B may not have provided sufficient training duration to induce similar short-term changes in F0.

Voicing control refers to the ability to self-regulate vocal behavior. To render constant vocal quality and loudness, speakers unconsciously balance vocal effort and comfort to maintain voice quality, especially in noisy environments^25^. Jitter, shimmer, and HNR are important acoustic parameters in voice analysis. All groups exhibited a T2 < T1 trend for jitter, but Experimental Groups showed statistically significant inter-stage differences, this is due to that straw phonation can optimize the voice quality. Moreover, only Experimental Group B showed a significant decrease after the intervention in the three groups, suggesting that post-VLT vocal rest followed by straw phonation more effectively improves jitter than vocal rest or training alone. Shimmer primarily represents the amplitude variation between adjacent cycles and mainly reflects the degree of voice roughness^37^. Significant differences were found for shimmer in the main effect of time and the interaction between condition and time, only in Experimental Group B was there a significant drop in shimmer from T2 to T1 and a significant decrease at T2 in the three groups. This finding may be attributed to two possible aspects. First, after extended voice use, during vocal rest, the vocal folds stopped vibrating and remained in an open and relaxed state with minimal energy consumption, ensuring normal activation of the vocal folds in subsequent phonation. Second, straw phonation can increase impedance to achieve better glottic impedance matching, allowing more of the vocal tract’s storage capacity to return to the glottis and reducing energy loss, which likely optimizes the mode of vibration to enhance the vocal amplitude^1,38^. HNR, the harmonic-to-noise ratio, represents the ratio of harmonic energy to noise energy. The stronger the periodicity of the voice signal and the higher the proportion of harmonic components, the higher the HNR value^37^. In the study, HNR increased at T2 in all groups, but significance was only found in Experimental Group B, highlighting the superiority of combined rest training protocols. At T2, Experimental Group B outperformed the control group in jitter, shimmer and HNR, likely due to rapid vocal fold muscle activation and optimized phonation initiation, which is consistent with Kang et al.^39,40^. While the specific contributions of combining vocal rest and straw phonation require further clarification, their synergistic effect appeared to be more beneficial than either method alone. Although the participants were healthy adults, the observed stabilization of acoustic parameters suggests improved vocal quality following simulated occupational loading, which may help maintain voice clarity and consistency during high-demand professional voice tasks.

CPP, derived from dual fast Fourier transforms^41^. Among cepstral measures, CPPcs (cepstral peak prominence in connected speech) has been shown to be sensitive to voice changes in individuals with vocal fatigue and can be used to identify fatigue-related voice quality changes associated with laryngeal function^23^. Cepstral peak prominence (CPP) can be extracted from both sustained phonation and connected speech without requiring direct computation of the fundamental frequency^42^, rendering it a more accurate acoustic parameter for describing precise voice measurement^43,44^. Murton, Hillman, and Mehta^42^ suggested a CPP cutoff values of 9.33 dB for continuous speech, the higher the CPP, the better the voice quality. In the present study, there was a significant main effect of time and interaction between condition and time, all groups showed increased CPP values at T1 compared with T0, reflecting compensatory phonatory adjustments aimed at maintaining voice quality during vocal loading. At T2, Experimental Groups A and B exhibited significant CPP increases versus T0, likely due to post-VLT vocal fold activation and the warm-up effect of straw phonation^11^. Both experimental groups surpassed the control group, as indicated by the increase in CPP, demonstrating that straw phonation, alone or in combination with vocal rest, can enhance voice recovery more effectively than traditional vocal rest alone. The reason for this outcome was that straw phonation increases supraglottal pressure and vocal tract inertance, thereby enhancing source–filter interaction. This acoustic loading may facilitate impedance matching through functional vocal fold adduction, which can improve glottal efficiency and reduce noise components in the acoustic signal^19^, as reflected by increased CPP.

Vocal fatigue is an individual-specific and multifaceted phenomenon that reflects both perceived fatigue and performance-related limitations arising from sustained vocal demand, integrating subjective vocal symptoms and functional changes in vocal performance over time rather than representing a single, well-defined physiological condition^45^. Consistent with this, prolonged phonation has been associated with increased vocal discomfort^46^. The VTD (lump in the throat) and total VTD scores in the three participant groups increased significantly at T1 after VLT, indicating the successful induction of vocal fatigue. During this VLT, such one hour of voicing time translated to approximately 720,000 vocal fold vibrations, assuming an average fundamental frequency of 200 Hz, during which vocal folds experience repetitive and intense contractions, and speakers have to maintain constant laryngeal muscle tension, possibly by employing additional motor units as the rest begin to fatigue^1^. This inevitably requires more effort to sustain phonation, leading to an increased PPE. Regarding the post-rest (T2) condition, the control group retained elevated VTD (lump in the throat), indicating the limited efficacy of brief vocal rest in alleviating these symptoms. This result is consistent with the findings of Whitling et al.^47^. In contrast, VTD (lump in the throat) and total VTD scores returned to the pre-VTL condition from T2 to baseline (T0) in Experimental Groups A and B. This improvement in voice may be attributed to the effect of straw phonation in optimizing glottal vibration patterns, reducing viscosity^1^, and thus enhancing efficiency^30^. Regarding PPE, there was a significant increase at T2 compared with T0 in the three groups, but a significant decrease from T2 to T1, demonstrating that all three interventions were effective for improving symptoms that may reflect in muscle tension. This also implies that attention should be paid to the fact that muscle tension resulting from prolonged loud phonation remains difficult to alleviate in a short time, as it may represent a high-risk factor for voice disorders. Although Experimental Group B showed higher PPE scores than the control group at T1, this difference likely reflected inter-individual variability in subjective vocal effort following vocal loading rather than an intervention-related effect. More importantly, intergroup comparisons at T2 revealed significantly lower VTD (lump in the throat) and PPE scores in both experimental groups versus the control group, with no differences between Experimental Groups A and B. This indicates that 5-minute and 10-minute straw phonation protocols yielded comparable benefits for alleviating the sensation of lumps in the throat and vocal effort, both better than vocal rest alone. As a matter of fact, Experimental Group A had higher VTD (Total) scores than Experimental Group B at T2, and this result indicated that a short period of vocal rest before straw phonation may enhance the recovery of vocal tract discomfort after prolonged vocal loading. After one hour VLT, the laryngeal muscles and vocal fold tissues remain in a state of residual fatigue and increased viscosity. Extending phonatory activity under such conditions may exceed the immediate recovery capacity of the vocal mechanism, thereby aggravating subjective discomfort. Thus reflected the importance of timing in voice training. As subjective vocal fatigue often precedes more persistent voice problems in individuals exposed to repetitive or prolonged vocal demands, reductions in perceived effort and vocal tract discomfort are particularly meaningful. Lower perceived vocal effort and reduced vocal tract discomfort may facilitate more efficient vocal recovery after intensive voice use, which is especially relevant for occupational voice users such as teachers who are frequently exposed to prolonged vocal demands.

The present findings should be interpreted within the context of acute vocal loading and immediate post-loading recovery. Although the study did not investigate long-term adaptation, the observed improvements suggest that straw phonation protocols may facilitate more efficient short-term recovery following intensive voice use. Occupational voice users, such as teachers and other professional speakers, are frequently exposed to repeated episodes of high vocal demand rather than isolated single events. In this context, optimizing immediate recovery after each loading period may be meaningful, as insufficient recovery between episodes may contribute to cumulative vocal strain over time. Accordingly, while the present data do not establish long-term preventive effects, they support the potential role of combined vocal rest and straw phonation as a practical recovery strategy for individuals engaged in high-demand voice use.

Although the participants were primarily young healthy adults and female, this demographic profile may partially reflect certain occupational voice populations, such as teachers, among whom females are highly represented^48^ and have been reported to exhibit a higher prevalence of voice problems and greater susceptibility to vocal fatigue during prolonged voice use^49^. Because prolonged high vocal demand is common among professional voice users^50^, the present findings may provide useful insight into post-loading vocal recovery strategies. Nevertheless, future longitudinal studies including a broader age range, practicing professional voice users, and individuals with vocal fatigue are warranted to determine whether straw phonation protocols can promote sustained vocal adaptation and reduce the incidence of voice disorders.

## Conclusion

Based on estimated marginal mean differences with 95% confidence intervals, straw phonation was associated with improvements in voice quality following vocal loading. The study further revealed that a 5-minute vocal rest followed by 5-minute straw phonation after prolonged vocal loading produced greater improvements in immediate post-loading vocal recovery. This protocol showed particularly notable effects in reducing the total VTD and PPE scores, with marked improvement in the VTD item related to the sensation of “lump in the throat”. Additionally, the combination of 5-minute vocal rest and 5-minute straw phonation appeared more effective than continuous 10-minute straw phonation in improving the total VTD scores, highlighting the importance of timing for performing straw phonation exercise after prolonged phonatory loading tasks. Overall, straw phonation may facilitate vocal recovery following prolonged voice use. A 5-minute vocal rest followed by a 5-minute straw phonation may represent a more effective strategy for promoting immediate post-loading vocal recovery. The supervised and standardized delivery of the intervention may further support the reproducibility and practical applicability of these findings.

## Data Availability

The data that support the findings of this study are not publicly available due to the privacy of the research participants, but are available from the corresponding author upon reasonable request.
